# Flexible Target Prediction for Quantitative Trading in the American Stock Market: A Hybrid Framework Integrating Ensemble Models, Fusion Models and Transfer Learning

**DOI:** 10.3390/e28010084

**Published:** 2026-01-11

**Authors:** Keyue Yan, Zihuan Yue, Chi Chong Wu, Qiqiao He, Jiaming Zhou, Zhihao Hao, Ying Li

**Affiliations:** 1 School of Data Science and Artificial Intelligence, Guangdong University of Finance, Guangzhou 510521, China; 2 Choi Kai Yau College, University of Macau, Macau SAR 999078, China; 3 School of International and Continuing Education, Beijing Institute of Technology (Zhuhai), Zhuhai 519088, China; 4 School of Computer Science and Artificial Intelligence, Foshan University, Foshan 528225, China; 5 School of Computer Engineering, Guangzhou City University of Technology, Guangzhou 510800, China; 6 Beijing Key Laboratory of Commercial Data Security Protection and Intelligent Governance, Beijing Technology and Business University, Beijing 100048, China; 7 Beijing Key Laboratory of Applied Statistics and Digital Regulation, Beijing Technology and Business University, Beijing 100048, China

**Keywords:** machine learning, quantitative trading, stock price prediction, American stock market

## Abstract

Stock price prediction is a core challenge in quantitative finance. While machine learning has advanced the modeling of complex financial time series, existing methods often rely on single-target predictions, underutilize multidimensional market information, and are disconnected from practical trading systems. To address these gaps, this research develops a hybrid machine learning framework for flexible target forecasting and systematic trading of major American technology stocks. The framework integrates Ensemble Models (AdaBoost, Decision Tree, LightGBM, Random Forest, XGBoost) with Fusion Models (Voting, Stacking, Blending) and introduces a Transfer Learning method enhanced by Dynamic Time Warping to facilitate knowledge sharing across assets, improving robustness. Focusing on ten key stocks, we forecast three distinct momentum indicators: next-day Closing Price Difference, Moving Average Difference, and Exponential Moving Average Difference. Empirical results demonstrate that the proposed Transfer Learning approach achieves superior predictive performance and trading simulations confirm that strategies based on these predicted momentum signals generate substantial returns. This research demonstrates that the proposed hybrid machine learning framework can mitigate the high information entropy inherent in financial markets, offering a systematic and practical method for integrating machine learning with quantitative trading.

## 1. Introduction

Interdisciplinary research integrating finance and computer science is advancing rapidly, with growing applications in areas such as option pricing and default risk modeling [1,2,3]. In quantitative finance, stock price forecasting represents a central line of inquiry, serving both to mitigate trading risks and to pursue excess returns [4]. From an information theory perspective, financial markets can be viewed as high-entropy systems shaped by continuous information flows and inherent uncertainties reflected in price movements. Consequently, predicting stock prices entails the identification of temporary, localized low-entropy signals within a broadly high-entropy environment [5,6]. Driven by improvements in computational hardware and declining model training costs, methodological approaches in this field have evolved from traditional statistical models toward advanced artificial intelligence techniques, particularly machine learning and deep learning algorithms [7]. These methods provide greater flexibility in capturing complex nonlinear dynamics present in financial time-series data [8]. Recent empirical studies underscore the effectiveness of hybrid frameworks that combine established time-series models with data-driven machine learningtechniques. For example, Generalized Autoregressive Conditional Heteroskedasticity (GARCH), along with its variants such as Glosten-Jagannathan-Runkle GARCH (GJR-GARCH) and Exponential GARCH (EGARCH), have been integrated with Support Vector Machines (SVM) to forecast financial indicators [9]. Moreover, optimization algorithms like Particle Swarm Optimization (PSO) have been successfully coupled with SVM or deep learning architectures to predict movements in equity indices such as the S&P 500 index [10]. Convolutional Neural Networks (CNN), originally developed for computer vision tasks, have also been merged with Long Short-Term Memory (LSTM) to form CNN-LSTM models, which have been applied to forecast carbon prices in Chinese markets [11]. These hybrid methodologies demonstrate considerable potential for improving predictive accuracy, thereby supporting the development of more profitable automated trading systems.

However, a significant limitation persists in the extant literature on stock price prediction: current research efforts remain concentrated on forecasting univariate targets, such as future price levels or short-term directional movements like next-day price changes [12,13]. Although such studies possess academic value, this narrow focus inherently restricts the exploitation of multidimensional information embedded within price dynamics, thereby constraining the breadth of analytical inquiry. Empirical evidence indicates that predictive performance varies substantially across different target variables, model architectures, and temporal horizons [14,15,16]. More critically, single-target prediction frameworks are often inadequate for capturing the multidimensional uncertainties stemming from the market’s inherent entropy. For example, high-frequency trading strategies exhibit sensitivity to immediate momentum signals like Closing Price Returns, while trend-following approaches rely on smoothed indicators more heavily, such as Moving Average Crossovers. As a result, a model optimized for predicting next-day Closing Price Differences may yield divergent or even contradictory outcomes when applied to strategies based on Moving Average (MA) Differences [17]. This disconnect between predictive modeling and strategic implementation fundamentally undermines the adaptability and robustness of quantitative trading systems.

This research introduces an innovative flexible target machine learning framework designed to enhance quantitative trading by simultaneously forecasting three variables: the next-day Closing Price difference, MA difference, and the Exponential Moving Average (EMA) difference. This flexible target setup establishes a robust analytical foundation for evaluating algorithm strategies. By capturing distinct temporal dynamics of market momentum, these complementary predictors enable more resilient quantitative decision-making. The framework is designed to measure market behavior across multiple dimensions, thereby encoding information from different levels of the price series more completely and offering a finer depiction of the market’s complex entropy structure. While Ensemble Models such as LightGBM and XGBoost exhibit strong standalone predictive performance, they often underutilize latent information available in cross-stock datasets. To address this limitation, we incorporate Transfer Learning technology [18], which extracts domain knowledge from source domains containing structurally similar stocks, thereby mitigating the high noise and uncertainty inherent in single-stock data.

We propose and validate a hybrid machine learning framework for flexible target stock prediction designed to support quantitative trading decisions. The methodology consists of three interconnected stages. First, we construct an extensive feature set derived from historical price data and technical indicators for ten American technology stocks: Apple (AAPL), Advanced Micro Devices (AMD), Amazon (AMZN), Broadcom (AVGO), Google (GOOG), Meta Platforms (META), Microsoft (MSFT), NVIDIA (NVDA), Oracle Corporation (ORCL), and Tesla (TSLA), selected based on their significant index weights and market capitalization dominance. Second, we define three distinct prediction targets: the next-day Closing Price Difference, MA Difference, and EMA Difference. To capture complementary market signals, each target is modeled using Ensemble Models (AdaBoost, Decision Tree, LightGBM, Random Forest, and XGBoost), Fusion Models (Voting, Stacking, and Blending), and Transfer Learning. Finally, trading strategies are systematically constructed from model predictions across stock, model, and target dimensions, and rigorously evaluated via backtesting in a simulated environment. Performance is assessed based on profitability, risk-adjusted returns, and robustness under varying market regimes. This research makes contributions in the following three aspects:

1. This research proposes a hybrid forecasting framework that integrates Ensemble Models, Fusion Models, and Transfer Learning for flexible target stock prediction, demonstrating superior performance compared to conventional single-target forecasting models. The framework offers investors a more comprehensive analytical foundation and yields predictions with broader practical applicability.
2. We illustrate how predictions for distinct targets like Closing Price Difference, EMA Difference, and MA Difference can be systematically translated into quantitative trading strategies. This establishes a clear and reproducible pathway from model outputs to executable trading logic, thereby offering actionable insights for real world quantitative decision making.
3. Through extensive empirical evaluation, we provide robust evidence on the comparative effectiveness of various machine learning models under flexible target settings and underscore the role of transfer learning in mitigating overfitting and enhancing predictive accuracy. These findings offer systematic support for employing machine learning techniques to better characterize and manage market uncertainties from an information theory standpoint.

The remainder of this paper is organized as follows: Section 2 reviews the relevant literature on stock prediction, machine learning techniques, and the application of Transfer Learning in finance. Section 3 delineates the methodological framework, encompassing data preprocessing, feature engineering, model specifications, prediction algorithms, and trading strategy design. Section 4 elucidates the experimental results derived from the model predictions, along with an analysis of the quantitative trading outcomes. Finally, Section 5 summarizes the key findings of the research and outlines potential directions for future research.

## 2. Related Works

This section synthesizes the evolution of methodologies in financial time series prediction, tracing the progression from traditional statistical models to contemporary machine learning and deep learning algorithms. We then examine a critical yet frequently underemphasized factor in stock prediction literature: the significant influence of target variable selection on model performance. Subsequently, we analyze recent advances and persistent challenges associated with applying Fusion Models and Transfer Learning within financial contexts. Finally, we conclude by synthesizing the limitations of prior research to clearly delineate the novelty and contributions of our research.

Financial time series prediction is a core in quantitative finance. Early research mainly relied on linear statistical frameworks, including Autoregressive Moving Average (ARMA), Autoregressive Integrated Moving Average (ARIMA), and Generalized Autoregressive Conditional Heteroskedasticity (GARCH) models, to forecast price movements and volatility under specific stationarity assumptions [19,20]. However, the inherent stochasticity, nonlinear dynamics, and non-stationarity of financial markets fundamentally limit the efficacy of these parametric approaches, which often struggle to capture the complex, chaotic characteristics of asset prices [21]. The advent of high-performance computing and big data infrastructure has catalyzed a paradigm shift toward data-driven, non-linear modeling techniques. Machine learning and deep learning algorithms, such as Random Forests, Neural Networks, LSTM, and Transformer with attention mechanisms, have emerged as state-of-the-art solutions due to their superior capacity for learning complex market patterns from data without restrictive prior assumptions [22,23,24]. A significant trend in current research involves the development of hybrid modeling frameworks that integrate knowledge across methodological domains. For instance, combining GARCH-family models with machine learning algorithms has demonstrated enhanced volatility forecasting performance and improved profitability across different stocks [25]. More recently, multimodal fusion strategies leveraging Large Language Models (LLM), Transformers, and CNN have been pioneered to harness cross-architectural synergies and mitigate prediction errors [26,27].

Existing literature on stock price prediction has primarily focused on forecasting directional movements or next-day Closing prices. However, it is increasingly recognized that the specification of target variables critically shapes input feature engineering and predictive accuracy, thereby exerting a decisive influence on the efficacy of quantitative trading strategies. For instance, Lim et al. demonstrated substantial performance heterogeneity across different temporal windows and label formulations in both machine learning and deep learning [28]. To mitigate noise interference, our previous paper introduced MA as alternative predictive targets. Empirical evidence confirms that applying MA transformations to price data can substantially reduce daily prediction errors [29]. This research extends the predictive scope from MA to include the EMA, constructing a more practically valuable forecasting framework. While MA-based predictions help identify and confirm stable trends due to their smoother response, the incorporation of EMA differences leverages its heightened sensitivity to recent price changes, enabling earlier detection of trend shifts and supporting short-term trading signals. Nevertheless, most existing studies remain confined to singular target paradigms, lacking systematic comparative analyses among Closing Price Difference, MA Difference, and EMA Difference within a unified modeling framework. At the modeling level, the field has evolved from single model systems toward hybrid architectures that integrate base learners through Ensemble Models.

Machine learning algorithms, including Random Forest, Gradient Boosting, SVM, and XGBoost have demonstrated strong predictive performance in equity markets by effectively controlling overfitting and capturing complex nonlinear feature interactions [30]. These methods, however, often rely on isolated ensemble assumptions, where each base model is trained separately on the same dataset. While effective in certain settings, this approach can lead to substantial performance fluctuations under different market regimes, thereby limiting prediction consistency and stability. To address this issue, Fusion Models have been introduced as integrated frameworks that combine multiple base models to enhance robustness and generalization capability beyond what a single model can achieve.Empirical support for such approaches extends beyond finance; for instance, in medical prognostics such as renal cell carcinoma, Fusion Models have been shown to yield more consistent predictions while retaining interpretability [31]. In quantitative finance, Gul et al. validated these advantages through backtesting on META stock, confirming that fusion-based methods deliver significantly improved stability in trading performance [32]. Furthermore, Transfer Learning offers a promising pathway to mitigate distributional shifts and data scarcity in financial time series analysis. By leveraging knowledge from source domains, such as historical data of established stocks or correlated assets, this approach enhances model adaptability for target domains with limited samples, such as newly listed equities [33,34]. Although widely adopted in computer vision, natural language processing, and robotics, the application of Transfer Learning in quantitative trading remains nascent [35,36]. This research explores the transfer of knowledge learned from American technology stocks, with the aim of improving predictive accuracy.

A predominant focus in extant financial forecasting research has been on predicting univariate targets, such as equity prices or crude oil futures, with evaluation often confined to point-prediction accuracy metrics. However, such studies often neglect to systematically integrate the predicted labels or signals into a complete quantitative trading framework for closed-loop validation [37,38,39]. We contend that bridging predictive analytics with executable strategy implementation constitutes an essential test of validity for any financial machine learning model. Our previous work has established foundational methodologies in this domain, demonstrating the efficacy of integrating regression-classification techniques into trading systems [40], as well as achieving robust performance through volatility-informed strategy design [25]. Building upon these advances, the present research extends the prediction targets from volatility measures to a set of multi-dimensional difference indicators, thereby enhancing market regime adaptability. Furthermore, we develop a backtesting environment that incorporates real world transaction constraints. The framework enables a rigorous evaluation of algorithm label synergies through professional performance metrics, including Annualized Return and Maximum Drawdown, thus establishing an integrated pipeline from predictive signal generation to decision support.

## 3. Methodology

**Figure 1:**
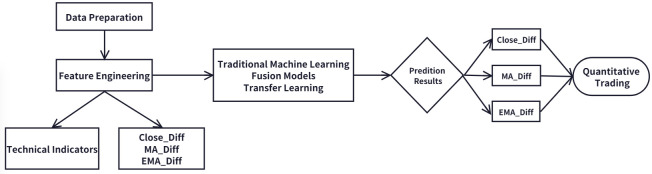
Experimental Framework.

This research establishes a systematic comparative framework to evaluate the predictive performance of Ensemble Models, Fusion Models, and Transfer Learning techniques in a flexible target price diffusion modeling context, focusing on high-profile American technology stocks. The resulting predictive signals are systematically integrated into quantitative trading strategies and rigorously evaluated through backtesting procedures. The overall technical architecture, illustrated in Figure 1, consists of three interconnected phases: data preparation and feature engineering, model construction and training, and quantitative trading strategy.

### 3.1. Data Preparation and Feature Engineering

This research selects a representative sample of ten leading technology stocks listed on the NASDAQ market: AAPL, AMD, AMZN, AVGO, GOOG, META, MSFT, NVDA, ORCL, and TSLA. These firms hold dominant positions in the global technology sector and exhibit high liquidity, substantial market capitalization, and pronounced sensitivity to market fluctuations, making them well-suited for evaluating predictive models. Daily market data spanning from January 2017 to December 2024 are obtained from official NASDAQ sources [41]. This period encompasses multiple market regimes, including periods of bullish momentum, high volatility, and sector-wide corrections, thereby providing a robust basis for model validation.

In contrast to conventional approaches that primarily focus on univariate targets such as directional price movements or Closing Price Differences, this research introduces an innovative flexible target prediction framework. It is designed to simultaneously capture complementary aspects of price dynamics through the following predictive objectives: the next-day’s Closing price Difference ($Close\_Diff_{t+1}$), the next-day’s MA Difference ($MA\_Diff_{t+1}$) and the next-day’s EMA Difference ($EMA\_Diff_{t+1}$). Among them, both MA and EMA are selected as the moving average values of the past five days. This flexible target design enables us to evaluate the relative performance of the model in predicting absolute price movements ($Close\_Diff_{t+1}$) and smoothing trend momentum ($MA\_Diff_{t+1}$, $EMA\_Diff_{t+1}$). The specific calculation formula for the dependent variable is as follows:

(1) $$Close\_Diff_{t+1}=Close_{t+1}-Close_{t},$$

(2) $$MA\_Diff_{t+1}=MA_{t+1}-MA_{t},$$

(3) $$MA_{t}\left(n\right)=\frac{Close_{t}+Close_{t-1}+\ldots +Close_{t-n+1}}{n},$$

(4) $$EMA\_Diff_{t+1}=EMA_{t+1}-EMA_{t},$$

(5) $$EMA_{t}\left(n\right)=\frac{2}{n+1}\sum_{i=0}^{t-1}{\left(\frac{n-1}{n+1}\right)}^{i}Close_{t-i}.$$

To quantify the uncertainties associated with the next-day $Close\_Diff_{t+1}$, $EMA\_Diff_{t+1}$, and $MA\_Diff_{t+1}$, we employ information entropy as the theoretical foundation for our calculations. Information entropy is a fundamental concept in information theory that measures the uncertainty inherent in a random variable [42]. For a discrete random variable X taking possible values $\left\{x_{1},x_{2},\ldots ,x_{n}\right\}$ with a corresponding probability distribution $P\left(X=x_{i}\right)=p_{i}$, the information entropy H(X) is defined as:

(6) $$H\left(X\right)=-\sum_{i=1}^{n}P\left(x_{i}\right)\log_{2}P\left(x_{i}\right).$$

The information entropy is measured in bits. A higher entropy value indicates greater uncertainty associated with the variable, which corresponds to a larger amount of embedded information and increased difficulty in predicting its fluctuation pattern. Conversely, a lower entropy reflects a more stable time series with reduced uncertainty. Among the three difference variables examined in this research, we assign a value of −1 when the difference is negative and 1 when it is positive. Following this binary discretization, the probabilities P(X=−1) and P(X=1) are derived, with their sum equaling 1. Based on the formal definition of information entropy, the entropy for each of the three price differences is computed, as summarized in Table 1. The results indicate that the entropy of the Close_Diff is higher than that of the EMA_Diff and the MA_Diff, implying greater predictive uncertainty. In contrast, the smoothed EMA and MA differences exhibit lower entropy and reduced uncertainty, providing a empirical rationale for selecting appropriate forecasting schemes based on different MA types.

The feature engineering framework employed in this research is designed to comprehensively capture information pertaining to market price trends, momentum, volatility, and trading volume. For each of the three dependent variables under consideration, we construct a set of trend and momentum attributes. These include Close, MA and EMA computed over multiple historical windows, which are widely adopted in financial forecasting to represent near-term, medium-term, and long-term price behaviors. While longer horizons such as the 50 days MA and 200 days MA are used as input variables [43,44], another recent paper incorporates averaged trading volumes as predictive features [45]. In our setup, the 5 days, 10 days, and 20 days MA or EMA are selected as inputs, corresponding to short-term, medium-term, and long-term trend signals commonly used in actual trading environments. Prior to feature construction, initial data cleaning is performed to remove missing values. In addition, to mitigate the influence of scale variation across variables, all numerical features are standardized using Z-score normalization, which enhances model stability and convergence during training.

1. For the Close_Diff dependent variable, we construct the Closing prices for the past five days ($Close_{t}$, $Close_{t-1}$, …, $Close_{t-4}$) and its daily price difference ($Close_{t}-Close_{t-1}$, …, $Close_{t-4}-Close_{t-5}$).
2. For the dependent variable MA_Diff, we construct the MA prices for the past five days ($MA_{t}$, $MA_{t-1}$, …, $MA_{t-4}$) including the consecutive 5 days MA, 10 days MA, and 20 days MA, and its daily degree difference ($MA_{t}-MA_{t-1}$, …, $MA_{t-4}-MA_{t-5}$).
3. For the dependent variable EMA_Diff, we construct the EMA prices for the past five days ($EMA_{t}$, $EMA_{t-1}$, …, $EMA_{t-4}$) including the consecutive 5 days EMA, 10 days EMA, and 20 days EMA, and its daily degree difference ($EMA_{t}-EMA_{t-1}$, …, $EMA_{t-4}-EMA_{t-5}$).

To construct a predictive model capable of comprehensively capturing market dynamics, we construct a set of financial technical indicators spanning multiple dimensions, including price trends, momentum, volatility, trading volume, and market sentiment to provide a unified representation of market conditions for all models. The selected features include Bollinger Bands, Relative Strength Index (RSI), Average True Range (ATR), On-Balance Volume (OBV), trading volume over the past four days, trading volume difference, Commodity Channel Index (CCI), Stochastic Oscillator (SlowK and SlowD), and Moving Average Convergence Divergence (MACD).

Bollinger Bands, a widely recognized volatility indicator developed by John Bollinger, consist of a central MA line flanked by an upper band and a lower band. These bands are positioned at a distance determined by the standard deviation of price, allowing the indicator to adapt dynamically to changing market volatility conditions. In practice, when the price touches the upper band, it is often interpreted as an overbought signal; conversely, touching the lower band is generally viewed as an oversold signal [46]. We incorporate both the upper and lower Bollinger Bands as input features. The calculations are performed as follows:

(7) $$Bollinger\;Middle_{t}=MA_{t}\left(20\right),$$

(8) $$Bollinger\;Upper_{t}=Bollinger\;Middle_{t}+2\times Std_{t}\left(Close,20\right),$$

(9) $$Bollinger\;Lower_{t}=Bollinger\;Middle_{t}-2\times Std_{t}\left(Close,20\right).$$

The RSI is used to measure the speed of price changes. It assesses whether an asset is in an overbought or oversold state by comparing the average increase and average decrease of the Closing price within a certain period [47]. The RSI range is from 0 to 100. It is generally believed that a value above 70 indicates overbought conditions and a value below 30 indicates oversold conditions. It is calculated using the standard 14-days cycle.

(10) $$RSI_{t}\left(n\right)=100-\frac{100}{1+\frac{\mathrm{average}\;\mathrm{gain}\;\mathrm{over}\;\mathrm{past}\;n\;\mathrm{days}}{\mathrm{average}\;\mathrm{loss}\;\mathrm{over}\;\mathrm{past}\;n\;\mathrm{days}}}.$$

The ATR indicator is used to measure the volatility of the market. It can be calculated based on the average difference of highest price and lowest price over a period of time, and can effectively capture the intensity of price fluctuations [48]. When the ATR value rises, it indicates increasing volatility; when the ATR value drops, it suggests that the market is calming down. It is calculated using a 14-days cycle.

(11) $$True\;Range_{t}=max(High_{t}-Low_{t},|High_{t}-Close_{t-1}|,|Low_{t}-Close_{t-1}|),$$

(12) $$ATR_{t}=MA_{t}\left(True\;Range,14\right).$$

OBV is a momentum indicator that combines trading volume with price changes. Its movement can be used to confirm the price trend or detect divergence. When the Closing price rises, the trading volume of the day will be added to the OBV. When the Closing price drops, the trading volume of the day will be reduced by OBV [49].

(13) $$OBV_{t}=\left\{\begin{array}{cc} OBV_{t-1}+Volume_{t}, & \mathrm{if}{\mathrm{Close}}_{t}>{\mathrm{Close}}_{t-1}\\ OBV_{t-1}, & \mathrm{if}{\mathrm{Close}}_{t}={\mathrm{Close}}_{t-1}\\ OBV_{t-1}-Volume_{t}, & \mathrm{if}{\mathrm{Close}}_{t}<{\mathrm{Close}}_{t-1} \end{array}\right.$$

CCI is used to determine whether asset prices have deviated. It compares the current price with the average price over a period and divided by the average deviation during that period [50]. The range of CCI usually varies between −100 and 100. Beyond this range, it may indicate a change in the strength of the trend.

(14) $$CCI=\frac{TP_{t}-MA_{t}\left(TP,n\right)}{0.015MD},$$

(15) $$TP_{t}=\frac{High_{t}+Low_{t}+Close_{t}}{3},$$

(16) $$MD=\frac{1}{n}\sum_{i=1}^{n}\left|TP_{i}-MA_{i}\left(TP,n\right)\right|.$$

SlowK and SlowD are momentum indicators that determine the strength of a trend and potential turning points by comparing the Closing price with the price range within a specific period of time. It consists of two lines: the fast line %K, and the slow line %D, which is the MA of %K [51].

(17) $$RSV_{t}\left(n\right)=100*\frac{Close_{t}-\mathrm{Lowest}\;{\mathrm{Low}}_{t}\left(n\right)}{\mathrm{Highest}\;{\mathrm{High}}_{t}\left(n\right)-\mathrm{Lowest}\;{\mathrm{Low}}_{t}\left(n\right)},$$

(18) $$SlowK_{t}=MA_{t}\left(RSV\right),$$

(19) $$SlowD_{t}=MA_{t}\left(SlowK\right).$$

MACD shows the direction, momentum, duration and intensity of a trend by calculating the difference between two EMA lines of different periods [52].

(20) $$MACD_{t}=EMA_{t}\left(12\right)-EMA_{t}\left(26\right).$$

### 3.2. Model Construction and Training

This section outlines the predictive modeling framework developed in this research, which is designed as a flexible target integrated learning system. The framework consists of three core components: Ensemble Models, Fusion Models, and Transfer Learning-enhanced methods. To ensure robust and comparable performance, five well-established machine learning algorithms: AdaBoost, Decision Tree, LightGBM, Random Forest, and XGBoost are selected as base models. These models span a range of structures from individual tree-based methods to ensemble techniques founded on distinct principles such as bagging and boosting, enabling the capture of a broad spectrum of potential linear and nonlinear patterns inherent in financial data [53]. To fully exploit the predictive capability of each model, hyperparameter optimization is systematically performed using a Grid Search strategy, with the principal objective of enhancing generalization performance. The detailed search spaces for the hyperparameters of each model are provided below.

1. AdaBoost: n_estimators (50, 100, 200, 300), learning_rate (0.001, 0.01, 0.1, 1).
2. Decision Tree: criterion (‘squared_error’, ‘friedman_mse’, ‘absolute_error’), max_depth (None, 10, 15, 20), max_features (None, ‘sqrt’, ‘log2’), min_samples_split (2, 5, 10), min_samples_leaf (2, 4, 8).
3. LightGBM: learning_rate (0.01, 0.05, 0.1), n_estimators (100, 200, 300), max_depth (3, 5, 7), reg_alpha (0, 0.1, 0.5), reg_lambda (0, 0.1, 1), feature_fraction (0.8, 0.9, 1.0).
4. Random Forest: n_estimators (50, 100, 200, 300), max_depth (None, 10, 20), max_features (‘auto’, ‘sqrt’, ‘log2’), min_samples_split (2, 5, 10).
5. XGBoost: n_estimators (25, 50, 100, 200), max_depth (3, 5, 8), subsample (0.6, 0.8, 1.0), reg_alpha (0, 0.1, 0.5, 1), reg_lambda (0, 0.1, 0.5, 1).

To integrate the strengths of diverse base models, this research implements three established Fusion Model strategies: Voting, Stacking, and Blending. For consistency and comparability, the same set of optimized Ensemble Models serves as the first-layer base models across all fusion approaches. In the Voting method, the regression predictions from all base models for future price differences are aggregated by computing their arithmetic mean, which is then adopted as the final fused output. The Stacking strategy employs a five-fold cross-validation procedure to generate out-of-fold predictions from the base models, which are subsequently used as meta-features. A Linear Regression model acts as the meta model to avoid overfitting problem, trained to optimally combine these predictions. For the Blending method, the training set is partitioned into a base model training subset and a held-out validation set. Predictions from the base models on this validation set form the meta-features used to train the meta model, reducing the risk of information leakage compared to Stacking.

The methodological innovation of this research lies in integrating Dynamic Time Warping (DTW) distance with Fusion Models, thereby introducing a Transfer Learning-enhanced ensemble approach for stock price prediction. In the context of time series prediction, DTW serves as a robust similarity measure that quantifies the alignment between sequences, even in the presence of nonlinear temporal distortions, thus enabling more informed knowledge transfer from source to target domains. By computing the optimal warping path between two sequences, DTW effectively captures similarities in shape, trend, and fluctuation patterns, which is critical for identifying suitable source domains from candidate datasets. Now DTW has been widely adopted in non-financial domains such as wind power prediction and energy forecasting [54,55,56]. And in stock prediction, DTW is also used to measure the similarity between different stocks for Transfer Learning [57]. In our research, DTW is employed to measure pairwise similarity among ten American technology stocks, all of which operate in related market segments and exhibit strong real world business linkages and correlated price movements. For each target stock, the DTW distances to the other nine stocks are computed based on their Closing sequences from 2017 to 2021. The resulting distance $d_{i}$ is used to assign a transfer weight $w_{i}$, defined as the reciprocal of the distance:

(21) $$w_{i}=\frac{1}{d_{i}}.$$

We introduce three integrated variants of Fusion Models combined with Transfer Learning: Transfer Voting, Transfer Stacking, and Transfer Blending. For each source stock i, a distinct Fusion Model is independently trained to predict a target variable specific to that source domain. This process yields a set of source-specific models $M_{i}$, each capturing domain-specific predictive characteristics. The final prediction for a target stock is obtained by computing a weighted aggregation of the predictions from all nine source-domain models. The weight $w_{i}$ assigned to each source model $M_{i}$ is determined by the inverse of the DTW distance between the source stock i and all the target stocks, thereby assigning greater influence to models originating from more similar market dynamics. The aggregated prediction is formally expressed as:

(22) $$Prediction\_final=\frac{\sum_{i}^{n}w_{i}\times M_{i}}{\sum_{i}^{n}w_{i}}.$$

This approach ensures that predictions from models trained on source stocks exhibiting higher dynamic pattern similarity with lower DTW distance are assigned greater influence in the final aggregated forecast. To evaluate the effectiveness of the proposed modeling frameworks, we compare the Transfer Learning-enhanced variants against two baseline categories: Fusion Models trained solely on the target stock’s data, and Ensemble Models. Predictive performance is quantified using four widely adopted regression metrics: the coefficient of determination (R Squared), Mean Absolute Error (MAE), Mean Squared Error (MSE), and Root Mean Squared Error (RMSE). These metrics collectively assess the deviation between predicted and actual values from complementary perspectives. The R Squared metric measures the proportion of variance in the dependent variable that is predictable from the independent variables. A value closer to 1 indicates a better model fit, meaning the model more effectively explains the variability of the target output. The MAE, MSE, and RMSE reflect different aspects of prediction error, with values closer to zero signifying higher accuracy. MAE provides a linear score of the average error magnitude, offering an intuitive interpretation. In contrast, MSE and RMSE, by squaring the errors, assign a disproportionately higher penalty to large prediction errors, thereby highlighting the model’s robustness to outliers. The formulas for calculating these metrics are provided below:

(23) $$R\;Squared=1-\frac{\sum_{t=1}^{n}{\left(y_{t}-{\hat{y}}_{t}\right)}^{2}}{\sum_{t=1}^{n}{\left(y_{t}-{\bar{y}}_{t}\right)}^{2}},$$

(24) $$MAE=\frac{1}{n}\sum_{t=1}^{n}|y_{t}-{\hat{y}}_{t}|,$$

(25) $$MSE=\frac{1}{n}\sum_{t=1}^{n}{\left(y_{t}-{\hat{y}}_{t}\right)}^{2},$$

(26) $$RMSE=\sqrt{\frac{1}{n}\sum_{t=1}^{n}{\left(y_{t}-{\hat{y}}_{t}\right)}^{2}}.$$

### 3.3. Quantitative Trading Strategy

Quantitative trading represents a systematic investment approach that relies on computational systems to execute automated trading decisions. By analyzing historical market data and identifying statistical patterns, this methodology aims to minimize the impact of investors’ subjective emotions, thereby improving investment efficiency and stability through disciplined strategy implementation. The innovation of the flexible target machine learning quantitative trading strategy proposed in this research lies in its comprehensive integration of multiple technical indicators to forecast short-term price movements, followed by rule-based trading operations derived from these predictions.

Innovatively, we employs three distinct price-based indicators: Close_Diff, MA_Diff and EMA_Diff as prediction targets to capture market momentum characteristics across multiple time horizons. Five Ensemble Models are utilized for prediction, and their outputs are integrated through Voting, Stacking, and Blending. To further enhance model robustness, Transfer Learning is incorporated to develop upgraded variants of each fusion approach. In total, eleven prediction models are constructed. Each model generates forecasts for the three target variables, yielding 33 distinct prediction results that collectively form a comprehensive quantitative trading signal. The trading strategy is designed as follows: a predicted positive difference for the next day is interpreted as a bullish signal, triggering a buy order when no position is held. Conversely, a predicted negative difference is treated as a bearish signal, prompting a sell order when a position exists. This logic is grounded in the assumption that a positive difference indicates strengthening momentum or an emerging uptrend, while a negative value suggests potential momentum decay or a trend reversal [58].

To evaluate the effectiveness of the trading strategy, we employ a backtesting framework to simulate real-market trading conditions. The simulation is implemented using the Backtrader platform, and the initial capital is $100,000, the commission for trading stocks is 0.025%, and slippage is also set. Annualized Return and Maximum Drawdown are selected as core performance metrics. The Annualized Return reflects the expected return of the strategy over a one-year horizon. A higher value indicates stronger profitability. It is calculated as follows:

(27) $$Annualized\;Return={\left(\frac{Asset_{end}}{Asset_{initial}}\right)}^{\frac{252}{n}}-1.$$

Maximum Drawdown measures the largest peak-to-trough decline in portfolio value during the investment period, serving as a key indicator of strategy risk. A smaller Maximum Drawdown implies better capital preservation and lower downside risk.

## 4. Experimental Results and Discussion

This section conducts a systematic performance comparison among Ensemble Models, Fusion Models, and Transfer Learning approaches for forecasting flexible target prices of major American technology stocks (AAPL, AMD, AMZN, AVGO, GOOG, META, MSFT, NVDA, ORCL, and TSLA). Based on the prediction outputs, quantitative trading strategies are formulated and assessed through backtesting. Predictive accuracy is evaluated using multiple regression metrics: R Squared, MAE, MSE, and RMSE. The dataset covers the period from January 2017 to December 2024. Daily trading data from 2017 to 2021 are used to train all models, while data from 2022 to 2024 are reserved as the test set for evaluating predictive performance and conducting quantitative strategy backtesting.

### 4.1. Regression Prediction Results

This section conducts a systematic comparative analysis of all combinations between machine learning models and predictor variables for each target stock. Specifically, three predictor variables are evaluated using eleven distinct machine learning algorithms, resulting in a total of 33 unique model-variable configurations. To ensure the robustness of the regression predictions, performance metrics are aggregated and summarized as the Mean plus or minus the Standard Deviation (Mean ± SD), providing an integrated evaluation of predictive consistency and stability across all stocks.

As illustrated in Table 2, the predictive performance exhibits considerable variation across individual stocks. Machine learning models achieve relatively high forecasting performance for AAPL, AMZN, AVGO, GOOG, NVDA, and ORCL, with MAE values consistently below 1.5, accompanied by comparatively low MSE and RMSE figures. In contrast, predictions for META, MSFT, and TSLA are associated with larger MAE values, exceeding 2.3. This performance disparity can be largely attributed to stock-level heterogeneity, wherein differences in market exposure, capitalization, liquidity, and firm-specific operational decisions collectively contribute to distinct price fluctuation patterns. Within the sample, stocks demonstrating smaller prediction errors, such as AAPL, AMZN, and GOOG, typically belong to well-established American technology giants characterized by substantial market capitalizations, and high liquidity. The price trends of these stocks tend to be relatively stable, less susceptible to singular unexpected events, and thus exhibit more consistent historical patterns that are more readily captured by machine learning algorithms. Conversely, each of the stocks associated with larger prediction errors presents unique challenges. TSLA is widely known for its high volatility and sensitivity to CEO commentary and shifts in market sentiment. These nonlinear and often difficult-to-quantify factors substantially increase forecasting difficulty. During the test period, META underwent a significant strategic transformation centered on its metaverse initiative, which introduced considerable valuation uncertainty and stock price fluctuations as the market reassessed its prospects. Although MSFT is generally regarded as a stable blue-chip stock, its price dynamics during the testing set were likely influenced by singular impactful events, such as its acquisition of Activision Blizzard. Such structural breaks can disrupt the statistical regularities present in historical data, thereby impairing the model’s ability to accurately capture subsequent price movements.

**Figure 2:**
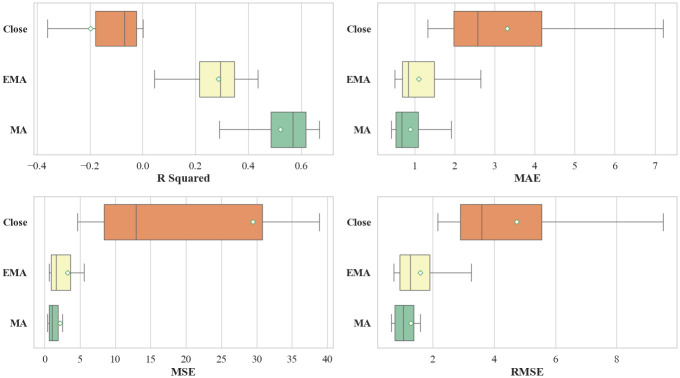
Comparisons of Regression Results Based on Dependent Variables.

We further classifies the results according to the dependent variable under consideration. As illustrated in Figure 2 and Table 3, the predictive performance varies substantially across the three target variables. These results underscore that the choice of prediction target exerts a decisive influence on the performance of machine learning models in financial time series forecasting. Among the three targets, the model predicting the Close_Diff exhibits the weakest performance. This is evidenced by a negative R Squared values and a larger SD relative to the other two targets. In terms of error metrics, the MSE for Close_Diff is not only significantly higher but also demonstrates considerable volatility. From a technical perspective, the EMA_Diff and MA_Diff incorporate smoothing that mitigates noise, thereby making underlying trends more discernible. In real market contexts, Closing prices are often susceptible to short-term noise and irrational trading behaviors, particularly during late trading sessions, which complicates trend extraction [59]. In contrast, EMA and MA offer a more robust representation of the genuine market trend by filtering out high-frequency fluctuations. Consistent with the entropy measures reported in Table 1, the higher entropy value associated with Close_Diff reflects greater underlying uncertainty, which in turn corresponds to the model’s inferior predictive performance.

In evaluating the performance of the machine learning models, Significant differences are observed across the different model categories. Ensemble Models contain AdaBoost, Decision Tree, LightGBM, Random Forest, and XGBoost. Fusion Models contain Voting, Stacking, and Blending. Transfer Learning contains Transfer Voting, Transfer Stacking, and Transfer Blending. As summarized in Table 4, the Transfer Learning approach achieves the highest average R Squared value, alongside the lowest mean values for MAE, MSE, and RMSE, indicating its superior overall predictive accuracy among the evaluated methods. The Fusion Models also demonstrate robust performance, with its evaluation metrics closely trailing those of the Transfer Learning Technique. In contrast, the individual Ensemble Models exhibit greater performance dispersion and relatively lower average predictive accuracy. The outstanding results of the Fusion Models and Transfer Learning suggest that strategies which effectively combine multiple base learners or leverage DTW based weighting for cross-domain knowledge transfer are better equipped to capture the complex, nonlinear dynamics inherent in flexible target stock price forecasting. The comparatively larger SD associated with the individual Ensemble Models’ results points to higher instability in performance across different stocks and target variables.

Table 5 provides a detailed breakdown of prediction errors for each model, based on the comparative framework established in Table 4. The results indicate that Ensemble Model, specifically AdaBoost and XGBoost exhibit moderate predictive performance, as reflected in their relatively low R Squared values and elevated MAE and RMSE. Furthermore, these models demonstrate considerable fluctuation across evaluation metrics, suggesting sensitivity to specific stock characteristics or market regimes. In contrast, Voting, Stacking, and Blending deliver more stable performance, with consistently lower error metrics and reduced variability. This aligns with the expectation that combining multiple base learners can mitigate individual model biases and enhance robustness. Notably, the Transfer Learning model achieves superior error control, slightly outperforming even the Fusion Models in terms of prediction accuracy. This improvement can be attributed to its ability to leverage shared patterns across related stocks, thereby reducing overfitting and strengthening generalization under market uncertainty. These findings are consistent with existing literature emphasizing the role of model integration and cross-domain knowledge transfer in improving forecast reliability in high entropy financial environments.

### 4.2. Quantitative Trading Results

**Figure 3:**
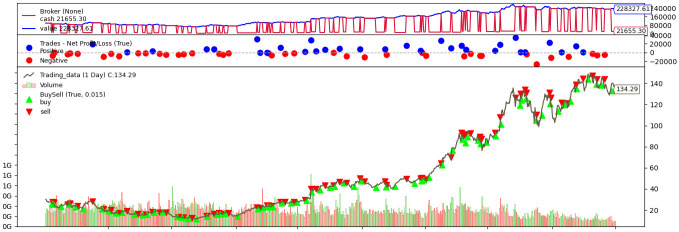
Trading Simulation of NVDA.

By classifying the trading performance at the individual stock level, we conduct a data analysis of strategy effectiveness. Figure 3 illustrates a simulated transaction sequence for NVDA using the Transfer Stacking method with the MA_Diff predictive target, where green markers indicate entry points and red markers denote exit points, visually reflecting profit and loss dynamics throughout the trading period. For each stock, we compute the Annualized Return and the Maximum Drawdown within a 95% confidence interval to ensure statistical robustness. Building on the prediction results outlined in the previous section, we select Fusion Models and Transfer Learning, representing the top-performing methodologies as the core engines for strategy construction. Correspondingly, the EMA_Diff and MA_Diff, which demonstrated lower prediction errors, are adopted as the primary forecasting variables in the quantitative trading system.

**Figure 4:**
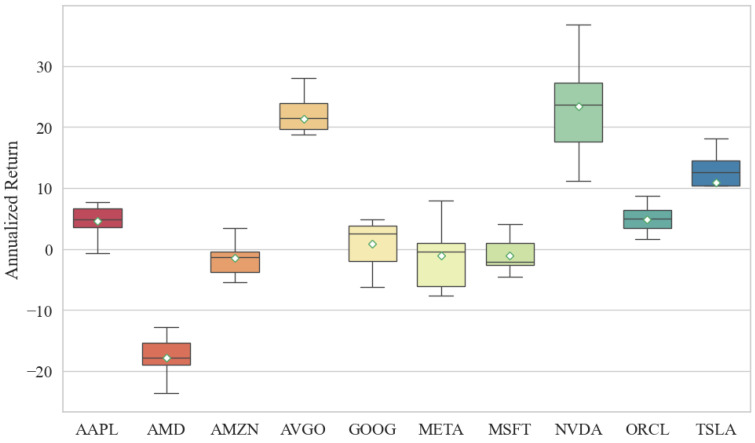
Comparisons of Annualized Return Based on Different Stocks.

As summarized in Table 6 and Figure 4, the machine learning driven strategies yield heterogeneous returns across the stock universe. Notably, AVGO, NVDA, and TSLA deliver the most pronounced profitability. In particular, AVGO and NVDA exhibit substantial cumulative gains, whereas TSLA, despite considerable fluctuations, also generates positive returns, a pattern conducive to buy-low-sell-high strategy modeling. Interestingly, although TSLA’s prediction accuracy was moderate in earlier evaluations, it achieves significant profitability in real world trading simulations, suggesting that prediction error alone does not fully capture trading potential. In contrast, other stocks such as AMD and META underperform. AMD consistently yields negative returns throughout the test period. Inspection of its historical price trajectory reveals that although its volatility is lower than that of high-momentum stocks like TSLA, it has largely oscillated within a bounded range without exhibiting a sustained upward trend, unlike NVDA. This limited directional movement likely explains the model’s inability to generate consistent profits in a volatile market regime. More critically, AMD contributes a relatively high Maximum Drawdown, which would substantially impact real world investor decision-making due to elevated risk exposure. While META’s returns within the 95% confidence band surpass those of AMD, its Maximum Drawdown level undermines its overall risk-adjusted performance, rendering it inferior to other technology equities.

Similarly, we evaluate the profitability performance of all stocks from a modeling perspective, as summarized in Table 7. In general, the Transfer Voting and Transfer Stacking methods outperform their non-transfer counterparts Voting and Stacking respectively. However, the standard Blending model exhibits superior performance compared to the Transfer Blending. Among all models, Transfer Voting achieves the most notable Annualized Return. Notably, the lower bound of its 95% confidence interval exceeds that of all other models, while the upper bound is also significantly higher. In terms of risk exposure, as measured by the Maximum Drawdown, the Blending model demonstrates the narrowest drawdown range, albeit at the cost of a relatively modest profit margin. For the remaining five model categories, there is no statistically significant difference observed in their Maximum Drawdown intervals. Therefore, when jointly considering risk-adjusted returns and drawdown control, we recommend prioritizing the Transfer Voting model for practical applications in quantitative trading.

We further classifies the quantitative trading outcomes according to the dependent variable under investigation. As illustrated in the Table 8, the trading performance of the model exhibits notable variation between the two target variables. The machine learning model based on the MA_Diff demonstrates marginally superior performance compared to the EMA_Diff, achieving an Annualized Return approximately 1–2% higher. In terms of risk control, the MA_Diff based model exhibits a Maximum Drawdown that is 1–3% lower, indicating better resilience under adverse market conditions. When considering the joint perspective of returns and risks, the MA_Diff focused strategy yields a more favorable risk-return profile.

Across both target variables, it is also observable that models incorporating Transfer Learning consistently outperform those relying solely on fusion techniques, with returns elevated by approximately 0.1–1%. This suggests that leveraging cross-stock knowledge transfer enhances predictive stability and profitability. In practical applications, investors may prioritize strategies built on MA_Diff, as they offer a more attractive trade-off between potential returns and risk exposure. Building on the insights from Table 7, investors are advised to consider adopting MA_Diff as the prediction target and employ Transfer Voting as the machine learning framework for stock forecasting, thereby constructing a robust and adaptive quantitative trading system.

## 5. Conclusions

This research presents a comprehensive evaluation of a hybrid forecasting framework that integrates Ensemble Models, Fusion Models, and Transfer Learning for flexible target stock price prediction and quantitative trading strategy generation in the American market. Through extensive experimentation and backtesting conducted on ten major American technology stocks, several key findings emerge. The proposed flexible target framework, simultaneously modeling the next-day Close_Diff, MA_Diff, and EMA_Diff, captures market dynamics more comprehensively than conventional single target forecasting approaches. Empirical results indicate that models predicting smoothed trend indicators such as MA_Diff and EMA_Diff achieve significantly lower forecasting errors compared to those targeting the raw Close_Diff series. This outcome underscores inherent differences among target variables in terms of noise resilience and trend representation. From an information theory perspective, the process of transforming raw price data into smoothed trends via technical indicators such as EMA and MA effectively filters out high-entropy market noise. The resulting sequences exhibit reduced complexity and a higher degree of temporal organization, which in turn enhances their predictability.

At the model level, Fusion Models consistently surpass individual traditional machine learning algorithms, underscoring the advantage of integrated Ensemble Models in enhancing prediction robustness and generalization capability. A key innovation introduced in this research, Transfer Learning further elevates performance by leveraging knowledge from stocks with analogous temporal patterns, thereby achieving the highest predictive accuracy in most situations. The results validate that Transfer Learning not only enhances target-stock forecasting by utilizing shared patterns from related equities but also effectively mitigates overfitting, particularly in high entropy market environments. Another major contribution lies in the direct translation of prediction outcomes into executable quantitative trading strategies, which are subsequently evaluated through rigorous backtesting simulations. The Transfer Learning models, particularly when applied to EMA_Diff and MA_Diff, not only exhibits lower forecasting errors but also delivers superior trading performance, demonstrating a consistent ability to preserve capital and capture returns under American market.

Despite these results, several limitations should be considered. This research primarily relies on technical indicators derived from historical price and volume data. Future work can enhance the model’s robustness by incorporating more diverse data sources, such as macroeconomic indicators, fundamental corporate data, news sentiment, and social media analytics, to construct a more comprehensive representation of market conditions. The current Transfer Learning framework utilizes DTW as the core similarity measure; subsequent research can explore more advanced metrics based on sophisticated feature engineering or multimodal learning to better capture cross-asset relationships. Furthermore, the Transfer Learning architecture itself offers avenues for refinement. Investigating dynamic source stock selection mechanisms and cross-market knowledge transfer, rather than relying on a fixed set of technologically similar stocks, could improve adaptability to evolving market regimes. Extending the framework beyond individual stocks to model rotations among Exchange-Traded Funds across different sectors, represents another significant direction for developing more diversified strategic portfolios.

In terms of modeling, subsequent research may explore advanced deep learning architectures, such as Attention, Transformers, and Temporal Convolutional Networks (TCNs), to better capture long-range dependencies and complex, non-linear patterns inherent in financial time series. Finally, the application of LLM in interpreting market events, generating explanatory narratives for predictions, and even synthesizing trading logic presents a promising frontier for enhancing both the performance and interpretability of quantitative trading systems.

In summary, the proposed hybrid framework introduces a novel and effective methodology for integrating machine learning with quantitative trading. Its practical utility has been empirically validated through backtesting, demonstrating its ability to construct localized, dynamically adaptive structures within the prevailing high entropy market environment, ultimately yielding returns that consistently exceed the benchmark. Future research is expected to broaden the model’s applicability and enhance its robustness, thereby contributing to the advancement of intelligent quantitative investment systems.

## Figures and Tables

**Table 1:** The Information Entropy for Dependent Variables.

Variables	Close_Diff	EMA_Diff	MA_Diff
H(X)	0.996946	0.982083	0.985346

**Table 2:** The Error Performance for Each Stock.

Stock Code	R Squared	MAE	MSE	RMSE
AAPL	0.280 ± 0.326	1.226 ± 0.768	3.671 ± 4.081	1.620 ± 1.023
AMD	0.255 ± 0.325	1.651 ± 1.012	6.885 ± 7.622	2.230 ± 1.383
AMZN	0.149 ± 0.567	1.491 ± 1.025	6.207 ± 7.882	2.030 ± 1.442
AVGO	0.131 ± 0.126	1.147 ± 0.601	5.401 ± 4.904	2.097 ± 1.002
GOOG	0.298 ± 0.274	1.078 ± 0.668	2.943 ± 3.192	1.459 ± 0.902
META	0.246 ± 0.271	3.124 ± 1.910	32.067 ± 34.620	4.835 ± 2.948
MSFT	0.245 ± 0.296	2.331 ± 1.436	12.464 ± 13.588	2.998 ± 1.864
NVDA	0.146 ± 0.149	0.790 ± 0.418	2.062 ± 1.979	1.277 ± 0.656
ORCL	0.008 ± 0.682	1.006 ± 0.762	3.181 ± 4.240	1.464 ± 1.019
TSLA	0.273 ± 0.366	3.873 ± 2.486	41.239 ± 47.527	5.357 ± 3.541

**Table 3:** The Error Performance for Dependent Variables.

Variables	R Squared	MAE	MSE	RMSE
Close_Diff	−0.197 ± 0.384	3.309 ± 1.861	29.431 ± 34.082	4.730 ± 2.657
EMA_Diff	0.286 ± 0.111	1.108 ± 0.605	3.259 ± 3.566	1.589 ± 0.855
MA_Diff	0.520 ± 0.130	0.897 ± 0.499	2.146 ± 2.359	1.292 ± 0.691

**Table 4:** The Error Performance of Machine Learning Models for Different Styles.

Model Style	R Squared	MAE	MSE	RMSE
Ensemble Models	0.158 ± 0.463	1.826 ± 1.671	12.478 ± 25.930	2.602 ± 2.389
Fusion Models	0.234 ± 0.304	1.733 ± 1.535	10.950 ± 21.349	2.490 ± 2.179
Transfer Learning	0.248 ± 0.295	1.720 ± 1.526	10.831 ± 21.122	2.475±2.169

**Table 5:** The Error Performance of Different Machine Learning Models.

Machine Learning	R Squared	MAE	MSE	RMSE
AdaBoost	0.094 ± 0.736	1.795 ± 1.710	13.562 ± 30.468	2.615 ± 2.592
Decision Tree	0.135 ± 0.316	1.889 ± 1.641	12.325 ± 23.501	2.656 ± 2.296
LightGBM	0.260 ± 0.245	1.694 ± 1.496	10.528 ± 20.755	2.445 ± 2.134
Random Forest	0.204 ± 0.402	1.793 ± 1.612	11.810 ± 23.428	2.547 ± 2.306
XGBoost	0.097 ± 0.433	1.957 ± 1.861	14.165 ± 29.891	2.746 ± 2.574
Voting	0.225 ± 0.332	1.749 ± 1.552	11.256 ± 22.314	2.509 ± 2.227
Stacking	0.237 ± 0.328	1.725 ± 1.537	10.886 ± 20.905	2.484 ± 2.172
Blending	0.241 ± 0.244	1.725 ± 1.515	10.707 ± 20.793	2.478 ± 2.137
Transfer Voting	0.238 ± 0.325	1.736 ± 1.542	11.109 ± 21.937	2.493 ± 2.212
Transfer Stacking	0.254 ± 0.311	1.711 ± 1.527	10.741 ± 20.731	2.463 ± 2.162
Transfer Blending	0.251 ± 0.242	1.714 ± 1.510	10.642 ± 20.672	2.468 ± 2.133

**Table 6:** Machine Learning Quantitative Trading Performance for Different Stocks.

Stock Code	Annualized Return	Maximum Drawdown
AAPL	[3.109, 5.945]	[19.980, 26.013]
AMD	[−19.847, −15.831]	[49.990, 53.565]
AMZN	[−2.941, 0.081]	[27.279, 32.463]
AVGO	[19.026, 23.532]	[30.120, 33.672]
GOOG	[−1.296, 2.889]	[33.338, 38.575]
META	[−3.739, 1.887]	[43.555, 56.865]
MSFT	[−2.407, 0.385]	[29.015, 33.476]
NVDA	[19.012, 27.949]	[43.135, 47.977]
ORCL	[3.719, 6.081]	[21.511, 23.193]
TSLA	[6.890, 14.313]	[37.278, 41.915]

**Table 7:** Quantitative Trading Performance for Machine Learning Models.

Strategy	Annualized Return	Maximum Drawdown
Voting	[−0.404, 10.002]	[31.472, 41.644]
Stacking	[−1.275, 10.034]	[31.872, 42.016]
Blending	[−0.991, 8.814]	[29.244, 40.137]
Transfer Voting	[0.576, 11.868]	[30.899, 40.731]
Transfer Stacking	[−0.868, 10.224]	[31.732, 42.031]
Transfer Blending	[−1.715, 8.394]	[32.305, 42.400]

**Table 8:** Quantitative Trading Performance for Different Dependent Variables and Machine Learning Models.

Variable	Machine Learning	Annualized Return	Maximum Drawdown
EMA_Diff	Fusion Models	[−0.799, 7.282]	[33.608, 41.716]
	Transfer Learning	[−0.341, 8.079]	[33.379, 41.307]
MA_Diff	Fusion Models	[0.835, 9.829]	[30.084, 38.864]
	Transfer Learning	[0.979, 10.295]	[31.650, 40.247]

## Data Availability

The data presented in this research are available on request from the corresponding author.

## Abbreviations

The following abbreviations are used in this manuscript:

Generalized Autoregressive Conditional Heteroskedasticity	GARCH
Glosten-Jagannathan-Runkle GARCH	GJR-GARCH
Exponential GARCH	EGARCH
Support Vector Machines	SVM
Particle Swarm Optimization	PSO
Convolutional Neural Networks	CNN
Long Short-Term Memory	LSTM
Moving Average	MA
Exponential Moving Average	EMA
Autoregressive Moving Average	ARMA
Autoregressive Integrated Moving Average	ARIMA
Large Language Models	LLM
Relative Strength Index	RSI
Average True Range	ATR
On-Balance Volume	OBV
Commodity Channel Index	CCI
Moving Average Convergence Divergence	MACD
Mean Absolute Error	MAE
Mean Square Error	MSE
Root Mean Square Error	RMSE
Artificial Intelligence	AI
Temporal Convolutional Networks	TCNs
